# Miscellany

**Published:** 1899-03

**Authors:** 


### MISCELLANY.

The Samuel D. Gross Prize.—The second Quinquennial prize of $1,000 under the will of the late Samuel D. Gross, M. D., will be awarded January 1, 1900, by the Philadelphia Academy of Surgery.

The conditions annexed by the testator are that the prize "Shall be awarded every five years to the writer of the best original essay, not exceeding 150 printed pages, octavo, in length, illustrative of some subject in surgical pathology or surgical practice, founded upon original investigations, the candidates for the prize to be American citizens.

It is expressly stipulated that the successful competitor, who receives the prize, shall publish his essay in book form, and that he shall deposit one copy of the work in the Samuel D. Gross library of the Philadelphia Academy of Surgery.

The essays, which must be written by a single author in the English language, should be sent to Dr. J. Ewing Mears, 1429 Walnut street, Philadelphia, before January 1, 1900. Each essay must be distinguished by a motto, and accompanied by sealed envelope bearing the same motto, and containing the name and address of the writer. No envelope will be opened except that which accompanies the successful essay. The committee will return the unsuccessful essays if reclaimed by their respective writers, or their agents, within one year. The committee reserves the right to make no award if the essays submitted are not considered worthy of the prize.

